# Opportunities and challenges for the use of deep brain stimulation in the treatment of refractory major depression

**DOI:** 10.1007/s44192-024-00062-9

**Published:** 2024-03-14

**Authors:** Prashin Unadkat, Joao Quevedo, Jair Soares, Albert Fenoy

**Affiliations:** 1 Elmezzi Graduate School of Molecular Medicine, Feinstein Institutes for Medical Research, Northwell Health, Manhasset, NY USA; 2 Department of Neurosurgery, Donald and Barbara Zucker School of Medicine at Hofstra/Northwell Health, Hempstead, NY USA; 3 Center of Excellence On Mood Disorders, Faillace Department of Psychiatry and Behavioral Sciences, McGovern Medical School, The University of Texas Health Science Center at Houston, (UT Health), Houston, TX USA; 4 Feinstein Institutes for Medical Research, Northwell Health, Manhasset, NY USA; 5 Department of Psychiatry, Donald and Barbara Zucker School of Medicine at Hofstra/Northwell Health, Hempstead, NY USA; 6 Department of Neurosurgery, Donald and Barbara Zucker School of Medicine, Feinstein Institutes for Medical Research, Northwell Health, 805 Northern Boulevard, Suite 100, Great Neck, NY 11021 USA

**Keywords:** Deep brain stimulation, Resistant depression, Medial forebrain bundle, Review

## Abstract

Major Depressive Disorder continues to remain one of the most prevalent psychiatric diseases globally. Despite multiple trials of conventional therapies, a subset of patients fail to have adequate benefit to treatment. Deep brain stimulation (DBS) is a promising treatment in this difficult to treat population and has shown strong antidepressant effects across multiple cohorts. Nearly two decades of work have provided insights into the potential for chronic focal stimulation in precise brain targets to modulate pathological brain circuits that are implicated in the pathogenesis of depression. In this paper we review the rationale that prompted the selection of various brain targets for DBS, their subsequent clinical outcomes and common adverse events reported. We additionally discuss some of the pitfalls and challenges that have prevented more widespread adoption of this technology as well as future directions that have shown promise in improving therapeutic efficacy of DBS in the treatment of depression.

## Introduction

Major Depressive Disorder (MDD) is globally one of the most prevalent psychiatric diseases, characterized by persistently low mood, anhedonia (inability to experience pleasure), feelings of guilt and worthlessness, cognitive and sleep disturbances [1]. In the United States alone this represents a major healthcare burden, with nearly 21 million adults having experienced at least one episode of MDD, nearly 66% of whom eventually require treatment [2].

Psychotherapy and pharmacological treatment have strongly remained first line treatments with selective serotonin reuptake inhibitors usually the first choice of medications [3]. However, even after adequate trials with different classes of antidepressants, a subset of patients (10–20% patients) fail to achieve adequate therapeutic effect [4]. The most common consensus is that after two adequate trials of antidepressants fail to provide adequate relief, the disorder is termed treatment resistant depression (TRD) [5]. Of note, a move to avoid any “therapeutic nihilism” and emphasize treatment resistance as a continuum rather than an absolute endpoint, the term “difficult to treat depression” has been proposed as an alternative to TRD for future clinical trials [6, 7]. However, for the purposes of this review we will continue to use TRD to avoid confusions on terminology.

After adequate drug trials have failed, patients are then considered for brain stimulation interventions. Electroconvulsive therapy (ECT) to the non-dominant hemisphere or to bilateral temporal or frontal lobes is the most effective stimulation treatment for TRD especially in patients with catatonia or with acute suicidality [8–11]. More recently, focused treatment with repetitive transcranial magnetic stimulation (TMS) has received FDA approval for TRD [12]. The most common target for stimulation is the dorsolateral prefrontal cortex using repetitive stimulation over multiple days and has shown to have significantly higher response and remission rates compared to sham stimulation in randomized clinical trials [10, 13, 14]. However, lack of definitive consensus on stimulation protocols and target sites have remained a source of variability in treatment outcomes [15].

The neurosurgical management of patients with severe depression has a long history, with ablative procedures gaining significant traction in the early nineteenth century. Procedures such as leukotomies, capsulotomies and cingulotomies initially showed promising results and rapidly gained popularity in the medical community [16–19]. However, rather indiscriminate use, lack of precision in targeting leading to variable outcomes and the advent of anti-depressant medications rapidly put these procedures out of favor [20]. A resurgence in surgical management for TRD came about in 2005 when the FDA approved the use of vagal nerve stimulation for the treatment of TRD (failed trials of > 4 anti-depressant drugs). However, results showed only a modest efficacy of about a 30% response rate after 12 months of treatment, with just under two-thirds of patients relapsing at 2 years [21, 22].

In the last two decades, there has been significant interest in using deep brain stimulation (DBS) for the treatment of TRD. Nearly three decades after the first case of chronic high frequency stimulation was used to successfully treat the motor symptoms of Parkinson’s disease, DBS has been used to treat a variety of movement, psychiatric, pain and seizure disorders [23–27]. The procedure involves the placement of electrodes into one or more deeper structures of the brain. These electrodes are then connected to a subcutaneous implantable pulse generator that delivers chronic stimulation to the target site. While the precise mechanisms of action of DBS are not known, high-frequency stimulation can modulate local and distally connected neuronal populations within a pathological brain network to produce the desired therapeutic benefit, similar to those seen with ablative procedures [23]. Optimal target selection (depending on the condition being treated and underlying pathological brain circuits), accurate lead placement and optimal stimulation parameter selection can radically affect the overall response to treatment [28, 29].

In this review, we will focus on the use of DBS for TRD. We review each of the different anatomical brain targets used for DBS, the rationale for selection, stimulation paradigms and subsequent therapeutic response and outcomes. We also discuss the current challenges that have limited widespread adoption of DBS therapy in TRD as well as the most promising future avenues in optimizing and improving the treatment of depression.

## General considerations

A number of physician- or patient-rated clinical scales in the management of depression have been developed to either diagnose, quantify the severity or evaluate treatment response [30]. Two of the most common scales reported in clinical trials include the Hamilton Depression rating scale (HAM-D) and the Montgomery-Asberg Depression rating scale (MADRS). HAM-D is a 17 point physician rated scale first described in 1960 and is one of the most popularly used instruments in clinical trials [31]. Due to questions being raised on its limited sensitivity to measure change with therapy, the MADRS was introduced as an alternative in 1979 as a more sensitive marker of treatment response and has also been used frequently to assess therapeutic effects [32]. Most of the studies discussed in this review will have used changes in either the HAM-D or MADRS or a combination of the two to assess treatment efficacy.

Treatment efficacy termed as ‘responders’ is usually the primary outcome measure for most DBS related studies and has been described as at least 50% decrease in HAM-D or MADRS scores from baseline. This 50% cutoff on either of the clinical rating scales is thought to be roughly equivalent and has been shown to correspond to a significant improvement in clinical symptoms [33]. Another common secondary outcome measure frequently used is that of ‘remission’. While the precise definition of this term varies, it generally is used to indicate an absolute score of less than 6–8 on HAM-D or 10–11 on MADRS [33, 34].

There is lack of a clear precise diagnostic test to differentiate the subtypes of depression. Inclusion criteria for patients enrolled for DBS interventions differed to some extent across studies discussed in this review and maybe a factor in the variability of outcomes. Additionally, patients had to have failed multiple modalities of treatment (e.g., psychotherapy, multiple classes of antidepressant medications and ECT) before being considered for DBS as a last resort. At this point, the disease process has proven to be resistant to most forms of treatment and thus limited our understanding of DBS efficacy in patients with less severe forms of depression.

Lastly, adverse events specific to stimulation at each of the anatomical sites have been discussed in the sections below. These are typically related to side effects of modulating the intended brain circuit (usually mitigated by optimizing stimulation parameters) or may be due to spread of current to surrounding tissue that is in proximity to the intended target (these can now be mitigated with newer electrodes that have ‘current steering’ capabilities [35]). However, more general DBS related risks remain, majority of which can be managed with conservative treatment. Only a few cases reach the point of requiring revision or explant and include intracranial hemorrhages, infections (intracranial or at the pulse generator site), lead breakages and pain (most commonly at the site of the lead extensions). These apply to DBS surgery in general and are not discussed with each anatomical target.

## Anatomical targets

### The subcallosal cingulate

Historically, the subgenual or subcallosal cingulate (SCC) was often the site for surgical lesioning in the treatment of medically refractory depression and emerged as the first structure (and to date the most common) targeted for DBS in patients with TRD.

*Rationale* Mayberg et al. described the SCC as a key node in the ventral compartment of a limbic cortical model of depression and this area has repeatedly been shown to have increased activity (and blood flow) in transiently induced states of sadness (in healthy subjects), in primary depression and is even downregulated with anti-depressant treatments [36].

**Fig. 1:**
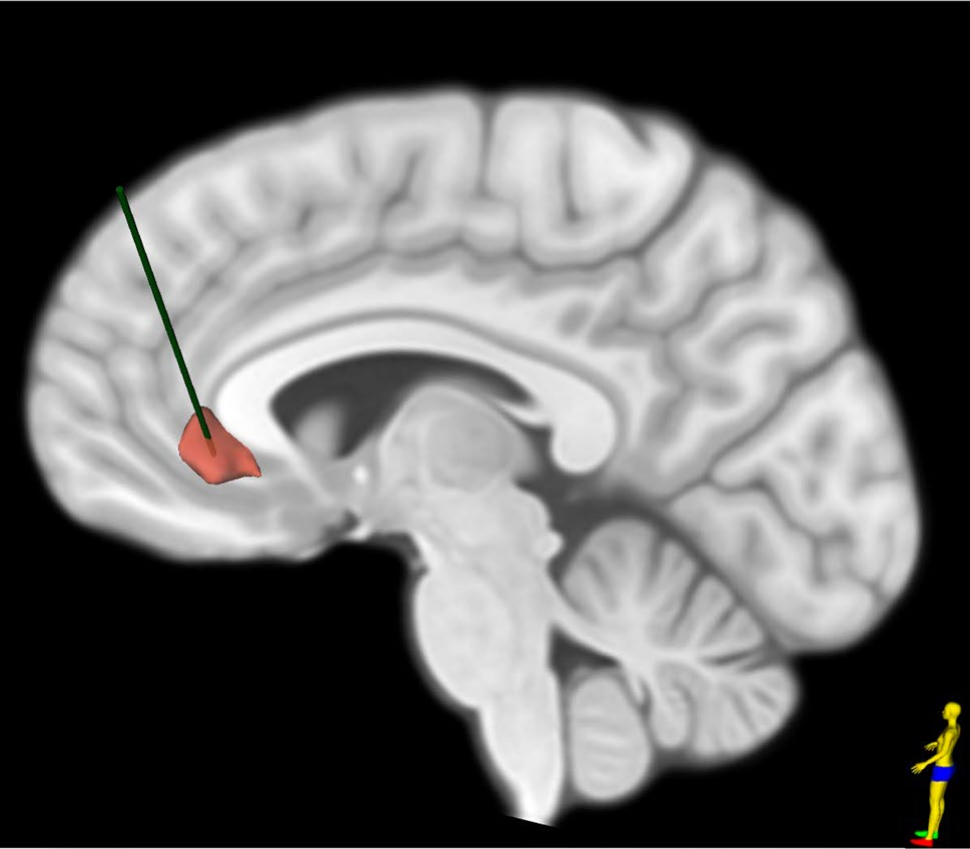
Sagittal view of a standard T1W brain MRI with a representative DBS electrode (green) within the subgenual cingulate cortex (SCC, red)

*Outcomes* In 2005, after over a decade of demonstrated efficacy of chronic DBS in the treatment of movement disorders, the first pilot study targeting the SCC enrolled six patients with treatment-resistant depression and showed sustained benefit in four out of the six patients, with PET imaging showing changes within key brain regions associated with depression [37]. Given these encouraging results, the authors enrolled a further 14 patients, observing both early and progressive improvements which were sustained at long term follow-up (up to 6 years) with an overall response rate of 55% at last follow up [38, 39]. These encouraging results demonstrated the safety and efficacy of SCC-DBS (Fig. 1) in the treatment of depression. With a response rate of roughly 60%, the alleviation of depressive symptoms and conferred therapeutic benefit was also associated with improved social reintegration (such as return to employment and more participation in leisure activities). Further, chronic stimulation was not associated with any significant cognitive side effects and only a few surgical related adverse events, non-specific to stimulation at the SCC. To address the apprehensions of stimulation related cognitive decline from disruption of SCC function in normal cognitive processing and memory tasks, Bogod et al. followed four patients after SCC-DBS upto 42 months and found that even though patients self-reported cognitive difficulties such as word finding difficulties, speech errors and short-term memory issues, no consistent declines in functioning were seen on a battery of neuropsychological tests within this cohort [40].

Holtzheimer et al. were the first to prospectively enroll 17 patients in study with a single blinded sham stimulation phase. Disappointingly, during the lead-in sham stimulation phase no significant differences were seen between postoperative and 4-week sham stimulation HAM-D scores. However, treatment in this cohort showed good long-term safety (2-years) and efficacy with only a few adverse events reported and a strong response and remission rate of 92 and 58% respectively, with an average reduction in HAM-D scores by about 70% [41]. More encouragingly, a second single blinded discontinuation phase after 24 weeks of active stimulation was abandoned after the first three patients had significant return of preoperative depressive symptoms and suicidality. Puigdemont et al. then followed up on responders and remitters from their prior study (n = 5) and entered them into a double blinded sham-controlled crossover study. At the end of the sham stimulation phase three out of the five patients experienced relapse of depressive symptoms, while active stimulation phase showed all patients in remission [42].

Similar response rates (both at short and long term) and therapeutic profiles have been observed at other sites with therapeutic benefit maintained at > 50% at 8 years [42–45]. However, concerns for low enrollment numbers and predominantly open label unblinded trials prohibited more widespread implementation.

The BROADEN trial to date is the largest study aimed to study efficacy of SCC-DBS. The study which began recruiting patients from 2008 was designed to be a 6 month, multi-center, randomized, double-blind, sham-controlled trial across 20 sites [46]. However, while initially aimed at enrolling 201 patients, the study was unfortunately cut short in 2012 after 90 patients were enrolled, with futility analysis demonstrating a low response rate without any significant differences between active and sham stimulation groups (20% vs 12%). Interestingly, post-hoc analysis on a subset of 77/90 patients showed response rates reached 49% and a remission rate of 29% at 24 months. More importantly, this improvement in efficacy was thought to be attributed to stimulation parameter changes which was not permitted during the initial study.

Up to this point all studies had electrodes targeted specifically to the SCC, however analysis from one group suggested that responders were patients that consistently showed active electrodes traversing surrounding white matter bundles [47]. In 2018, Riva-Posse and colleagues prospectively enrolled patients in an aim to investigate if implanted electrodes guided by white matter maps into areas of the SCC (specifically targeting four white matter bundles that connect to key nodes of a depression brain network) led to improved outcomes [48]. The authors demonstrated this with a response rate of 82% at 1 year. These results and other similar studies have shifted the field from an anatomical targeting approach to one that is connectomic-based to maximize clinical efficacy.

Successful SCC-DBS is dependent on several factors. Appropriate patient selection and precise targeting (to both the SCC and surrounding white matter) remain important considerations. However, the impact that stimulation parameter selection can have on the efficacy of treatment have often been overlooked. Follow-up results from the BROADEN trial reaffirmed this and demonstrated significant increase in response rates after stimulation optimization. Much work needs to be done in this field, as only a handful of studies have systematically assessed the impact that parameter selection (such as frequency and pulse width) can have on response rates [29, 49, 50].

*Summary* The SCC was the first anatomical area targeted specifically for depression and has most frequently been studied across multiple clinical sites. Underwhelming results from the BROADEN trial somewhat dampened the enthusiasm in targeting this area for TRD. However, recent observations in improved efficacy with the recruitment of surrounding white matter structures within the stimulation field as well as the potential for future patient specific stimulation parameter selection has led to resurgence of interest in its potential as a therapeutic target.

### Ventral capsule/ventral striatum

The ventral capsule (VC) is a white matter bundle that projects from the midbrain dorsally to frontal areas of the brain. The ventral striatum (VS), specifically the nucleus accumbens (NAc), plays a key role in the modulating reward, motivation and mood due to its connectivity with other nodes within the limbic circuitry [51].

*Rationale* Stimulation studies of the VC in humans as early as the 1970’s had shown emotional responses in schizophrenic patients [52]. Historically, these areas were often used as targets for ablative procedures in obsessive–compulsive disorder (OCD) [53]. Eventually, as an alternative to lesioning, neurosurgeons targeted this same area with DBS for the treatment of OCD. Imaging studies identified widespread connectivity from this region to areas that have abnormal activity in diseased patients [54]. Interestingly, the topography of this circuit has multiple overlapping regions with those implicated in depression such as the orbitofrontal cortex, the prefrontal cortex and the anterior cingulate cortex. Not surprisingly then, concomitant observations of mood elevation and even hypomania [55, 56] were seen in a subset of patients being treated for OCD. These findings gave rise to the interest in potentially using this target for the treatment of medically refractory depression.

**Fig. 2:**
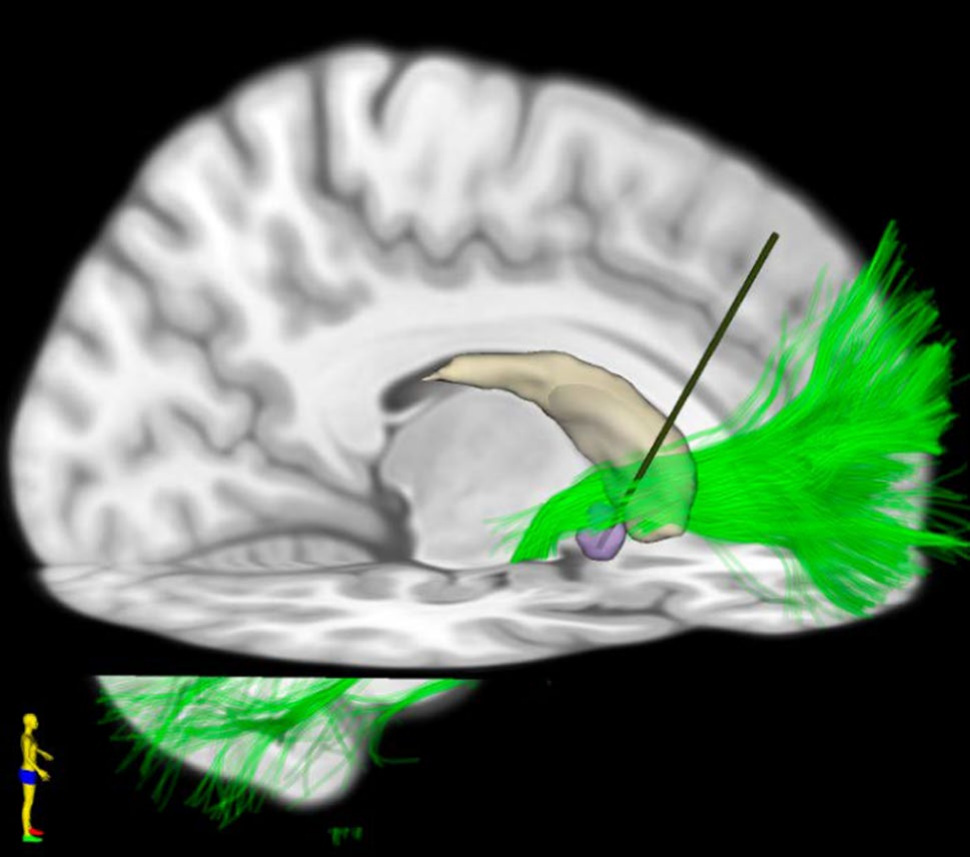
Sagittal view of a standard T1W brain MRI with a representative DBS electrode (green) traversing the anterior limb of the internal capsule (ALIC, bright green) and the nucleus accumbens (NAC, red) located ventral to the caudate (white)

*Outcomes* One of the first reported studies looking at DBS to this area (Fig. 2) was by Schlaepfer et al. They reported on results from three patients by targeting the NAc. Immediately upon stimulation patients experienced behavioral responses that were consistent with reward seeking. Subsequently long-term monitoring showed bi-directional improvements and worsening of depressive symptoms with randomized alternation of stimulation conditions (ON and OFF) [57]. Another open label study in 2009 included 15 patients across three sites [58]. Results showed an overall response rate of 53% at last follow up with about 40% of patients in remission. Shortly after, Bewernick et al. implanted 11 patients with electrodes in the ventral striatum (specifically the NAc). Half of the patients responded to treatment at 12 months with decreases in depression (specifically decreases in anhedonia) as well as an anxiolytic effect [59]. Follow up at 2 years, showed a stable response rate with one patient in continued remission [60]. Another study reported on a small cohort of patients (n = 4) enrolled in an open label trial. Initial results in the first 5 months of NAc stimulation showed some anti-depressant effects but none of the patients reached response or remission status. This was followed by 4 months of stimulation of the caudate nucleus by utilizing the more dorsal contacts on the electrodes without much success. Subsequently, an extension period where stimulation parameters were optimized showed two out of the four patients with significant response and another with mood fluctuations frequently meeting response criteria [61]. Given these promising results, the RECLAIM trial, a prospective multicenter randomized double blinded sham-controlled trial, began enrolling in February 2009 with a target of 208 patients [62]. Unfortunately, similar to the BROADEN trial targeting the SCC (described earlier), interim futility analysis after 30 patients did not show significant differences between active and sham stimulation groups. Overall, there was a responder rate of only 23.3% at 2 years, significantly lower than reports from previous open label studies; in part attributed to suboptimal study design leading to insufficient stimulation optimization phase. To the contrary, around the same time, a study out of the Netherlands showed more encouraging results. The authors specifically targeted the anterior limb of the internal capsule (ALIC) with the lowest contact ending in the NAc and enrolled 25 patients in a randomized double blinded crossover trial, after completing 52 weeks of an optimization phase. 40% of patients were deemed responders and had significantly lower depression rating during active stimulation compared to the sham (none of the patients were responders during the sham stimulation phase) [63]. A subset of patients additionally completed a maintenance phase (18 patients) and had long term follow up. Overall, stimulation had a response rate of 32% which increased up to 40% after stimulation optimization with overall stable levels of therapeutic response during the maintenance phase [64]. Along a similar theme, a group out of Beijing evaluated 10 patients with VC/VS stimulation for TRD. While the focus of the study was to identify structural and functional correlates from active contacts (discussed later) and reported differences in response to a multitude of stimulation paradigms, the cohort exhibited strong anti-depressant effects with an overall reduction of 55.8% in HAM-D scores [65].

Sustained long-term efficacy for this target has also been reported for smaller subsets of patients from the RECLAIM trial (n = 8). The authors found that while it took nearly 7 years for half the cohort to meet stable response criteria, anti-depressant effects were clearly stronger in those patients that continued with stimulation treatment (vs those that discontinued stimulation). Overall, treatment efficacy persisted in this group even 11 years out from surgery in 5/8 patients, exhibiting an overall reduction of 63% in MADRS scores from baseline [66]. Similarly, the Netherlands group also found stable response rate of 44% at last follow up (> 4 years) with the ratio of strong responders (> 75% HAMD-D decrease) to clear responders (50–75% HAMD-D decrease) continuing to increase even years after treatment. Improvements in depression severity with active stimulation also coincided with improvements in quality of life metrices that were not seen during sham stimulation periods [67].

Most common reported side effects with stimulation with the VC/VS include hypomania, mania and, in some cases, worsening anxiety (although with optimal settings stimulation at this site has been associated with anxiolytic effects) which typically is resolved with stimulation titration (typically decreases in voltage).

*Summary* DBS to the VC/VS has primarily been a target for OCD but has shown some promise in the treatment of TRD. Acute stimulation regimens have led to increased social behaviors and energy often in conjunction with improvements in mood [68]. Therapeutic response is noticeable within weeks of stimulation onset and is sustained after steady state of stimulation, with some patients even reporting acute worsening of symptoms when stimulation is withdrawn. Optimal location for implantation of the electrode within this large brain area varies to some extent across studies and may factor into the differences in outcomes across cohorts. Lack of efficacy compared to sham controlled patients from the RECLAIM trial has similarly dampened early enthusiasm, but interest continues to remain. The recent advent of non-invasive thermal lesioning using MRI guided focused ultrasound has particularly re-invigorated the possibility of targeting this area as an alternative treatment to DBS in carefully selected patients [69].

### Medial forebrain bundle (MFB)

The MFB and more specifically the superolateral branch is a major white matter bundle that connects various cortical and subcortical structures involved with the brain’s motivation and reward circuitry [70].

*Rationale* Projecting from the ventral tegmental area (VTA), the MFB projects to the NAc, prefrontal (PFC) and orbitofrontal cortices bilaterally [71]. This white matter bundle is mainly made up of excitatory glutaminergic fibers that connect the PFC to the VTA and subsequently within this same bundle send dopaminergic outputs from the VTA anterior and dorsally (in part through the ALIC, which in itself is a target for neuromodulation and discussed in the previous section) to various limbic and cortical areas involved in reward seeking and motivation [72]. Symptoms of depression such as anhedonia and dysphoria are thought to arise from dysfunction within this circuitry and the MFB provides a gateway to rapid modulation of this network with promising pre-clinical results from bilateral stimulation in animal models of depression [73, 74].

**Fig. 3:**
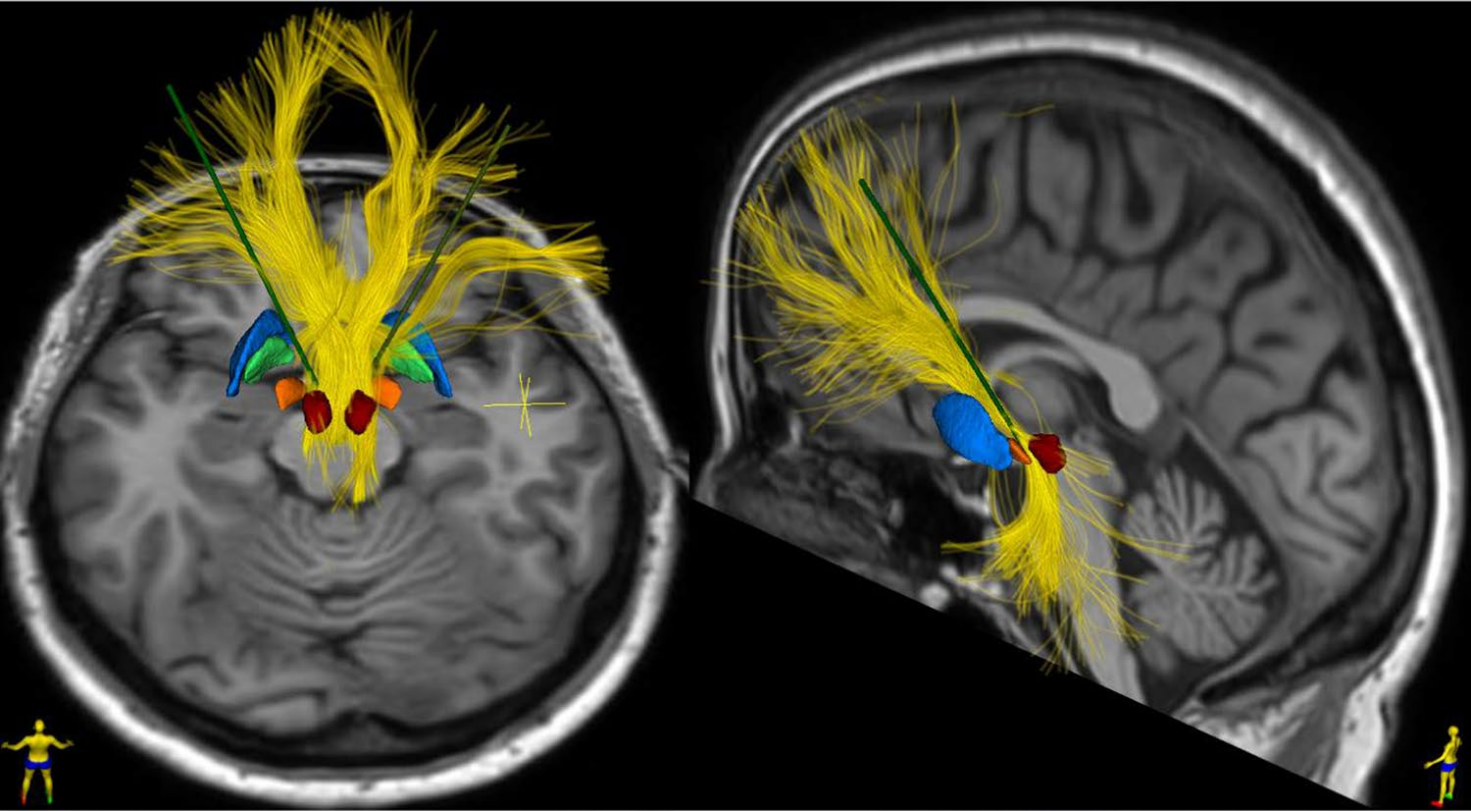
Axial and sagittal view of a T1W brain MRI from a 58-year female with TRD with bilateral electrodes (green) placed in the medial forebrain bundle (MFB, yellow). UKF two-tensor tractography model [127] is used to isolate the MFB and electrodes are stereotactically placed directly within the white matter bundle. The MFB is located medial to the subthalamic nucleus (orange) and anterior to the red nucleus (Red). Significant anti-depressant effects were seen post-DBS at 1 year with reduction in MADRS scores by 66% from preoperative baseline

*Outcomes* The MFB has only more recently become the target for DBS (Fig. 3) and was one of the first depression targets solely described by white matter tractography methodology [75]. The first report of the antidepressant effects of MFB stimulation found pronounced antidepressant effects (> 50% decrease in depression ratings) in 6 out of 7 patients. More impressively, these effects were seen in as little as 1 week of stimulation onset (unlike other targets that typically require weeks to months of stimulation to reach therapeutic levels) and were sustained until the 4-year follow up [76, 77]. Our group subsequently corroborated these results, with the initial experience of six patients demonstrating a > 70% decrease in MADRS rating at 1 year follow-up [78, 79].

Besides the effect of stimulation at the target, there appears to be a “microlesional” therapeutic effect possibly due to the inflammatory cascade that ensues following insertion of the electrode [80, 81]. Acute behavioral responses following electrode placement have been reported with insertion at other sites as well [82, 83]. However, these effects appear to be more consistently seen with MFB DBS, as evidenced by majority of patients demonstrating acute anti-depressant effects early on, however the prognostic value of this phenomenon as they relate to long term outcomes is still unclear [81].

In order to better decipher this insertional effect from stimulation induced changes, our group enrolled 10 patients with TRD in a single blinded sham-controlled study [84] and as expected, there were acute decreases in MADRS scores (29% decrease) during sham stimulation phase. However, a much larger therapeutic benefit was seen (averaging nearly 50% decreases in MADRS from preoperative period) after only 2 weeks of stimulation, with five patients classified as responders. Half the cohort completed their 5-year follow up, with four out of five patients continuing to show therapeutic response with long term average decrease in MADRS of nearly 81% [85]. Further, using ^18^F-Flurodeoxyglucose PET, we identified significant metabolic changes within the mediodorsal thalamus, caudate, cingulate cortex and PFC in response to stimulation with decreases in caudate metabolism specifically correlating with the therapeutic effects quantified via MADRS scores [86].

The largest study to date to study the effects of MFB-DBS come from the GATEWAY trial which was designed as randomized controlled onset of stimulation trial that enrolled a total of 16 patients with TRD [87]. Most patients reached response criteria after 1 week of stimulation. All patients reached response criteria (> 50% decrease in MADRS scores) during the study and half of the patients were in remission (< 10 on the MADRS scores) at 12 month follow up. Sani et al. interestingly studied one patient after MFB DBS using daily assessments that substantiated the general temporal trend of anti-depressant efficacy with a significant decrease in symptoms at 6 months [88]. While still a relatively a newer target, most studies have shown robust anti-depressant effects after stimulation; one report of two patients at a single center, however, did not demonstrate any therapeutic efficacy in either patient with unknown reasons for failure, although lack of descriptive methodology in target determination limited interpretation. The authors did not use microelectrode recording or autonomic responses during intraoperative stimulation that may have also  contributed to failure of treatment [89, 90].

All reported studies encountered only minor adverse events. Transient oculomotor symptoms (diplopia) were most often reported and related to stimulation spread that resolved with adjustments in settings. One study did report good therapeutic benefit after MFB stimulation but unexplained sudden onset of blurry vision 9 months after implantation that did not resolve with stimulation adjustment, eventually requiring selection of a different target [91]. Also, one case of hypomania that resolved with parameter change has also been reported, however this appears to be a rare side-effect and we did not observe any cases of hypomania in our cohort.

*Summary* The MFB has only in the last decade been used as a therapeutic target for TRD. Results from these initial cohort of patients suggest MFB DBS has the potential to have a sizeable anti-depressant effect with relatively rapid onset that appear to be sustained even at 3–5 years after surgery.

### Other targets

#### Lateral habenula (LHB)

**Fig. 4.**
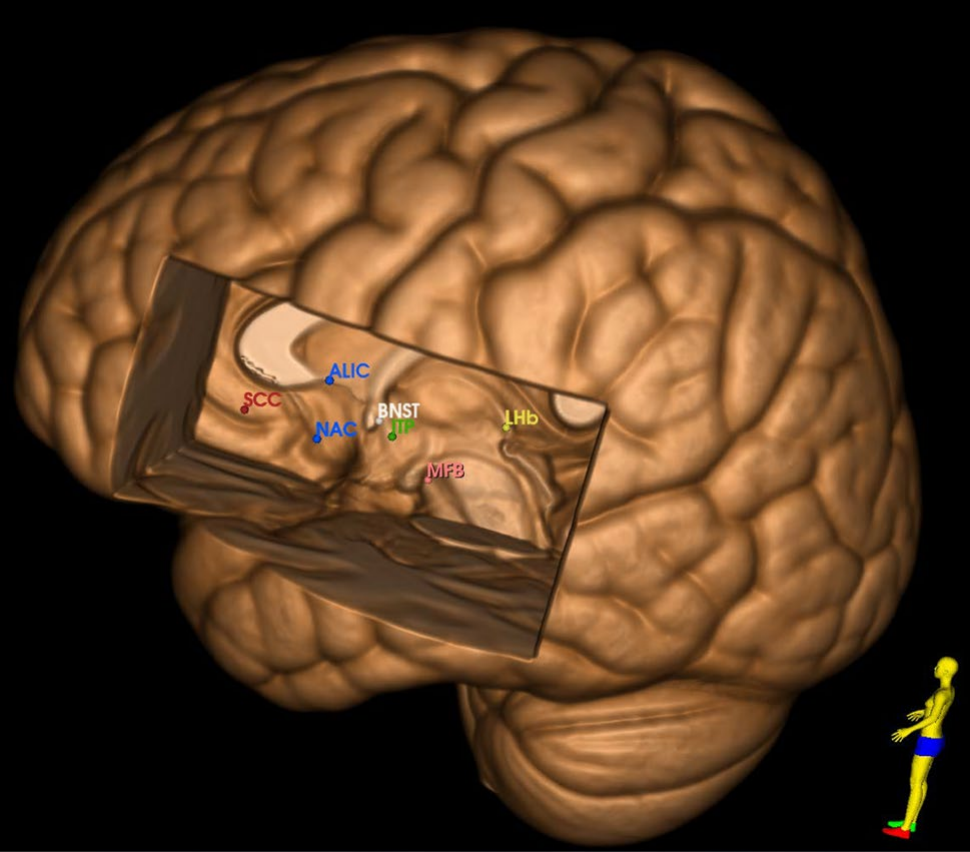
3D rendering of a standard brain template with all DBS targets. *SCC* subgenual cingulate cortex, *BNST* bed nucleus of stria terminalis, *ITP* inferior thalamic peduncle, *MFB* medial forebrain bundle, *NAc* nucleus accumbens, *ALIC* anterior limb of the internal capsule, *LHb* lateral habenula

The LHB is a small epithalamic nucleus (Fig. 4) that has been found to be hyperactive in animal models and patients with depression and has been implicated in encoding negative-valence signals and promoting behavioral aversion [92]. Rather infrequently targeted for DBS, small studies have shown some promise in targeting LHB for treatment of depression and bipolar disorder [93–96]. The first report that investigated the efficacy of bilateral LHB demonstrated remission of severe depression after stimulation and found return of symptoms after accidental discontinuation of stimulation [93]. The largest series to date included seven patients with bipolar disorder or depression and showed modest response (64% reduction in depression scores), however results were limited by the small sample size and high dropout rate [95].

#### Inferior thalamic peduncle (ITP)

The ITP originates from the interlaminar thalamic nuclei traversing its way through the ventral capsule to the prefrontal cortex (Fig. 4). The first report of ITP stimulation in a single patient demonstrated strong anti-depressant effects after which the patient entered a double blinded sham stimulation phase that resulted in significant fluctuations in depression ratings normalized again by re-initiation of stimulation [97]. Following this, a small study compared stimulation of the ALIC vs ITP in a two stage crossover design [98]. However, no significant difference in response and remission rate were found between the two targets. However noteworthy is that only one patient subjectively preferred ITP stimulation over ALIC.

Most common adverse events with this target included a transient increase in depressive symptoms and suicidal ideation. One patient did have extrapyramidal symptoms during stimulation that disappeared when the stimulator was turned off [98].

#### Bed nucleus of stria terminalis (BNST)

The BNST is small complex structure that is located posterior to the NAc (Fig. 4) and participates in certain types of anxiety and stress responses, receiving inputs from the amygdala and relays them to the hypothalamus and brainstem nuclei [99]. One study compared local field potential activity within this structure between patients with depression and OCD and found exaggerated alpha band activity within this region in the depression cohort [100]. A single patient report found a gradual but significant improvement (52% decrease in MADRS scores) with stimulation at the stria terminalis [91]. Shortly after an open label study with five female patients found good response rate with three out of five patients meeting response criteria at 12 month follow up [101]. Like the first report, therapeutic relief was gradual with only one patient responding to stimulation at 6 months. Two patients did have a suicide attempt however they were non-responders at the time of the event (one of those patients never reached response throughout the follow up period). Given the limited experience and small cohort of patients treated with stimulation at this target, much work needs to be done to establish anti-depressive efficacy of DBS at this site.

All reviewed studies discussed are summarized in Table 1 and targets are displayed in Fig. 4.

**Table 1:** Reviewed studies by anatomical target and commonly reported stimulation-related side effects

Study	Year	No. of subjects	Study design	Rating scale	Responder rate	Remission rate	Stimulation related adverse events
SCC							Increased depression, anxiety, sleep disturbances
Mayberg [19]	2005	6	Open label	HAM-D	66%	50%	
Kennedy [20]	2011	20	Open label	HAM-D	55%	35%	
Holtzheimer [23]	2012	17	Open label (sham lead-in phase)	HAM-D	92%	58%	
Puigdemont [24]	2012	8	Randomized controlled cross-over trial	HAM-D	62.50%	50%	
Lozano [25]	2012	21	Open label (multicenter)	HAM-D	29%	–	
Merkl [26]	2013	6	Randomized sham control trial (double blinded)	HAM-D	33%	33%	
Ramasubbu [32]	2013	4	Randomized (stimulus optimization phase)	HAM-D	50%	25%	
Bogod [22]	2014	4	Open label	HAM-D/MADRS	50%	–	
Holtzheimer [28]	2017	90	Randomized sham control trial (double blinded)	MADRS	49%	26%	
Eitan [31]	2018	9	Randomized controlled cross-over trial (high vs low frequency stimulation)	MADRS	44%	–	
Riva-Posse [30]	2018	11	Open label	HAM-D	81.8%	54.6%	
Ramasubbu [33]	2020	22	Randomized cross over trial	HAM-D	23%	27%	
VC/VS							Worsening depression, hypomania
Schlaepfer [40]	2008	3	Open label	HAM-D/MADRS	0%	0%	
Malone [41]	2009	15	Open label	HAM-D/MADRS	53.30%	40%	
Bewernick [42]	2010	10	Open label	HAM-D	50%	30%	
Bewernick [43]	2012	11	Open label	HAM-D	45%	9%	
Millet [44]	2014	4	Open label	HAM-D	75%	25%	
Dougherty [45]	2015	30	Randomized	MADRS	23.30%	20%	
Bergfeld [46]	2016	25	Open label	HAM-D	40%	20%	
Van der Wal [47]	2020	25	Open label	HAM-D	44.40%	27.80%	
Hitti [63]^a^	2021	8	Open label	MADRS	50%	25%	
Lai [62]	2023	10	Open label	HAM-D/MADRS	–	–	
MFB							Visual disturbances, abnormal eye movements
Bewernick [56]	2017	8	Open label	MADRS	75%	50%	
Sani [64]	2017	1	Open label	CAT-DI/HAM-D	100%	100%	
Fenoy [58]	2018	6	Open label (single blinded sham stimulation phase)	MADRS	67%	66.70%	
Coenen [63]	2019	16	Randomized controlled (onset of stimulation)	MADRS	100%	50%	
Davidson [65]	2020	2	Open label	HAM-D	0%	0%	
Fenoy [61]	2022	10	Open label (single blinded sham stimulation Phase)	MADRS	80%	–	
LHb							Visual disturbances, dizziness, mania, impulsivity
Sartorius[69]	2010	1	Open label	HAM-D	100%	100%	
Zhang [70]	2019	1	Open label	HAM-D	100%	100%	
Wang [72]	2021	1	Open label	HAM-D	100%	0%	
Zhang [71]	2022	7	Open label	HAM-D	42.90%	14.30%	
ITP							Increased depression, sleep disturbances, extrapyramidal symptoms
Jimenez [73]	2005	1	Open label	HAM-D	100%	100%	
Raymaekers [74]	2017	7	Randomized (double blind crossover trial)	MADRS	57.1% (IC/BST); 42.9% (ITP)	28.6% (both)	
BNST							Insomnia, anxiety, tearfulness, involuntary smiling, physical discomfort
Blomstedt [67]	2017	1	Open label	HAM-D	100%	0%	
Fitzegerald [77]	2018	5	Open label	HAM-D	60%	60%	

*SCC* subgenual cingulate cortex, *BNST* bed nucleus of stria terminalis, *ITP* inferior thalamic peduncle, *MFB* medial forebrain bundle, *NAc* nucleus accumbens, *ALIC* anterior limb of the internal capsule, *LHb* lateral habenula

^a^Cohort are a subset of patients reported in Dougherty et al. [45] that had long-term follow up

## Discussion: challenges and opportunities

Since the first report of DBS implantations for depression was described in 2005, several groups have demonstrated feasibility of alleviating the symptoms of depression in an extremely difficult to treat cohort of patients. In all studies, DBS was used after patients had failed responses to medications and trialed other modalities with multiple rounds of ECT and psychotherapy. At this point, these patients are left with no other options but to continue cycling through combinations of treatments that have already failed. Despite the challenges of TRD, most studies regardless of the anatomical target and design show that response rates after DBS are superior to the natural course of TRD. With continued standard of care, less than 20% of these patients would be projected to show any response after 2 years, and only 8% of these patients would be expected to be in remission [102]. While larger randomized control trials were prematurely stopped for a lack of therapeutic response, smaller carefully treated cohorts have shown clear therapeutic efficacy. Work in the last two decades have provided several insights about depression as a disease process as well as highlighted potential opportunities to improve treatment paradigms with DBS.

Firstly, evidence suggests depression is a heterogenous syndrome with a broad range of pathological brain circuits implicated rather than a single disease state [103]. Naturally then, it is conceivable why one treatment strategy may not work for all patients. Neuroimaging studies have attempted to characterize these syndromes to enable more specific treatment based on the underlying pathology; however, this is still not a routine clinical reality in its present state [104, 105]. Dysfunction in both positive and negative valence systems in the brain have been implicated in the pathogenesis of depression and either one can predominate resulting in different phenotypes of the same clinical diagnosed disease [106]. The VTA, NAc, and the orbitofrontal cortex are key components within the positive valence system that regulates reward seeking behaviors, motivation, and engagement. Functional MRI studies have found these areas to be downregulated in depressed patients [107]. Naturally then, DBS electrodes implanted in areas (such as the VC/VS and MFB) that modulate the positive valence system may potentially benefit those patients that predominantly have anhedonia and hopelessness [108]. On the other hand, negative valence systems control responses to anxiety, fear and loss and include areas such as the amygdala, striatum and insula and are upregulated in depressed patients [109]. Patients who have maladaptive responses within this system may then benefit preferentially from other targets such as the SCC [83].

Secondly, improvements in target localization on a patient-specific basis have improved the efficacy of DBS treatments. Modern neuroimaging techniques using diffusion imaging based tractography can provide optimal areas for electrode implantation to enable precise modulation of pathological circuits and is more specific than a co-ordinate-based site selection [110]. A landmark paper in this field by Riva-Posse at. al demonstrated an *82% responder rate* when prospectively enrolling patients for SCC-DBS that was guided by white matter mapping; a *significant* improvement to prior studies with the same target [47]. When analyzing our cohort of patients with MFB DBS we found that those patients that had a higher structural connectivity from the implantation site to the OFC had much higher anti-depressant responses [86]. Similarly, modulation of fiber bundles traversing near electrode contacts in the ventral VC/VS and connecting to the lateral and medial prefrontal cortices have been shown to improve response rates and tend to predict a better improvement [65]. Such findings are not limited to DBS for psychiatric disorders only. Tractography based targeting has also shown its utility in improving outcomes for more routine indications such as for movement disorders. Recruitment of the hyperdirect pathway and the dentato-rubro-thalamic tract within the volume of activated tissue around the DBS electrodes can improve outcomes in both Parkinson’s Disease and Essential Tremor [111, 112]. Not only is this useful for targeting, but understanding the relationship of the implanted lead to the surrounding white matter can aid the clinician in selecting the optimal stimulation parameter and electrode contact [113]. With an increasing number of centers implementing these technique into their routine pre- and post-surgical planning, it is clear that a connectomic-based approach is fundamental to reducing variability in operative outcomes [114].

Thirdly, numerous studies have found significant improvements in treatment response rates with optimization of stimulation paradigm [29, 46, 49, 50, 64]. It is now abundantly clear that a one-size-fit-all approach leads to a suboptimal treatment effect and has been a major reason for failure of some of the studies described above. Currently, stimulation paradigms are chosen during postoperative visits using a trial-and-error method, with patients reporting subjective changes in their mood and perception to guide the clinician. However, without a reliable objective metric that a clinician can measure (such as tremor or rigidity for movement disorders), modifications for psychiatric conditions can be a rather slow and somewhat subjective process, especially since many DBS treatments take weeks to months of constant stimulation to provide meaningful therapeutic benefit. More importantly, periods of non-clinically significant mood fluctuations may cloud the overall trajectory of improvement experienced by the patient, prematurely pushing the clinician to make changes to stimulation parameters and potentially resetting the clock to reach stable therapeutic responses. This has driven the need towards trying to identify biomarkers of treatment efficacy that may precede the clinical benefit that is apparent to the patient. Broadway et al. found early changes in frontal-theta cordance recorded from resting state scalp EEG to predict more delayed improvements in depression severity after SCC-DBS [115]. Others have tried to use intracranial recordings during surgery with good success as well. In one such study, intracranial local field potential changes (LFP) (more specifically in beta power within the left hemisphere) during surgical implantation and after brief SCC stimulation predicted short term improvements in depression severity and correctly classified responders [116]. Another group identified local field potential changes that temporally reflected the severity of depressive symptoms in a single patient [117]. A more significant breakthrough towards identifying a clinically meaningful biomarker was recently reported in a small cohort of patients implanted with SCC electrodes [118]. Using long-term longitudinal LFP recordings from the site of implantation, the authors were able to use an electrophysiological (EP) marker based neural classifier to correctly identify depressed and treatment response states in individual patients with very high accuracy (AUC ~ 87%) which even demonstrated sensitivity to changes in stimulation. While prospective validation in this study was limited to one out-of-sample patient, the EP marker impressively demonstrated the possibility to identify impending relapses and need for intervention (such as stimulation parameter changes) almost a month before this was measurable by current clinical instruments. Similar approaches in identifying biomarkers specific to DBS interventions have also been reported for other disorders such as in chronic pain [119]. These results hold the promise for more objective titration of therapy for the clinician and thus can be expected to reduce the variability in response rates for future clinical trials.

Lastly, due to the heterogeneity of depression as a pathological disease process, some have chosen an approach that requires developing individualized treatment plans and subsequently identify biomarkers of therapeutic efficacy on a patient-by-patient basis. To this end, attempts have been made to use intracranial EEG recordings and neurobehavioral testing to then guide ultimate DBS treatment, more akin to what is routinely done in epilepsy surgery [120–122]. An intracranial recording approach is certainly appealing and adds to a network- level understanding of the dysfunction underlying the different phenotypes of depression and is likely necessary in the short-term. However, replication of this strategy on a patient-by-patient basis, requiring multistage procedures and extended in -hospital monitoring may prohibit the larger scale implementation of DBS in routine clinical practice. More ideally, then, would be to use a non-invasive (perhaps image-guided) stratification of disease phenotype and selection of treatment based on the predicted response. To this end, Drysdale et al. found stratification of depression into four biotypes that were defined by distinct patterns of functional connectivity [123]. More impressively, they were able to use this to predict response to TMS therapy. On a similar accord, it is feasible to consider that selection of a DBS stimulation site may be chosen based on a similar functional imaging stratification. In our own cohort of MFB DBS patients, we have potentially identified a ^18^F-FDG PET based metabolic network that was modulated in response to MFB stimulation, with preoperative network activity predictive of the magnitude of postoperative anti-depressant effects after stimulation [124].

## Future opportunities

Two decades of work have collectively shown powerful anti-depressive effects in cohorts of patients with DBS. However, consistent responses in larger-scale studies are still lacking and are fundamental for DBS to be considered as a mainstream therapy for TRD. It is becoming abundantly clear that personalized treatment strategies are needed to reduce the variability in response after DBS. Pre-operative patient stratification, followed by an individual connectomic-guided surgery and an artificial intelligence optimized stimulation paradigm selection are all being actively investigated and may be a plausible reality in routine clinical practice in the not too distant future. With the addition of sensing capabilities (the ability to record local field potentials) at the electrode site, the future of DBS therapy looks more likely to reflect a closed loop and adaptive stimulation strategy with wireless recording capabilities providing insights into depressive brain network fluctuations in naturalistic settings outside the operating room. This can then be used to trigger or titrate the stimulation when electrophysiological markers suggest an exaggerated depressive state [125, 126].

## Conclusion

The introduction of DBS as a treatment option for depression has offered an alternative for those in whom standard therapies have failed with few treatment options remaining. While still an off-label use, studies have shown robust anti-depressive responses in small groups of patients. Newer strategies have focused on improving patient selection as well as personalizing treatments tailored to the individual patient that holds the promise to improve as well as homogenize the variability in therapeutic response.

## Data Availability

Not applicable.
